# Ascertainment of Minimal Clinically Important Differences in the Diabetes Distress Scale–17: A Secondary Analysis of a Randomized Clinical Trial

**DOI:** 10.1001/jamanetworkopen.2023.42950

**Published:** 2023-11-15

**Authors:** Jack Banks, Amber B. Amspoker, Elizabeth M. Vaughan, LeChauncy Woodard, Aanand D. Naik

**Affiliations:** 1 Department of Management, Policy and Community Health, School of Public Health, University of Texas Health Science Center, Houston; 2 Institute on Aging, University of Texas Health Science Center, Houston; 3 Houston Center for Innovations in Quality, Safety, and Effectiveness, Michael E. DeBakey Veterans Administration Medical Center, Houston, Texas; 4 Department of Internal Medicine, Baylor College of Medicine, Houston, Texas; 5 Department of Internal Medicine, University of Texas Medical Branch, Galveston; 6 Tilman J. Fertitta Family College of Medicine and Humana Integrated Health Systems Sciences Institute, University of Houston, Houston, Texas; 7 Department of Internal Medicine, McGovern Medical School, University of Texas Health Science Center, Houston

## Abstract

**Importance:**

The Diabetes Distress Scale–17 (DDS-17) is a common measure of diabetes distress. Despite its popularity, there are no agreed-on minimal clinically important difference (MCID) values for the DDS-17.

**Objective:**

To establish a distribution-based metric for MCID in the DDS-17 and its 4 subscale scores (interpersonal distress, physician distress, regimen distress, and emotional distress).

**Design, Setting, and Participants:**

This secondary analysis of a randomized clinical trial used baseline and postintervention data from a hybrid (implementation-effectiveness) trial evaluating Empowering Patients in Chronic Care (EPICC) vs an enhanced form of usual care (EUC). Participants included adults with uncontrolled type 2 diabetes (glycated hemoglobin A_1c_ [HbA_1c_] level >8.0%) who received primary care during the prior year in participating Department of Veterans Affairs clinics across Illinois, Indiana, and Texas. Data collection was completed in November 2018, and data analysis was completed in June 2023.

**Interventions:**

Participants in EPICC attended 6 group sessions led by health care professionals based on collaborative goal-setting theory. EUC included diabetes education.

**Main Outcomes and Measures:**

The main outcome was distribution-based MCID values for the total DDS-17 and 4 DDS-17 subscales, calculated using the standard error of measurement. Baseline to postintervention changes in DDS-17 and its 4 subscale scores were grouped into 3 categories: improved, no change, and worsened. Multilevel logistic and linear regression models examined associations between treatment group and MCID change categories and whether improvement in HbA_1c_ varied in association with MCID category.

**Results:**

A total of 248 individuals with complete DDS-17 data were included (mean [SD] age, 67.4 [8.3] years; 235 [94.76%] men), with 123 participants in the EPICC group and 125 participants in the EUC group. The MCID value for DDS-17 was 0.25 and MCID values for the 4 distress subscales were 0.38 for emotional and interpersonal distress and 0.39 for physician and regimen distress. Compared with EUC, more EPICC participants were in the MCID improvement category on DDS-17 (63 participants [51.22%] vs 40 participants [32.00%]; *P* = .003) and fewer EPICC participants were in the worsened category (20 participants [16.26%] vs 39 participants [31.20%]; *P* = .008). There was no direct association of DDS-17 MCID improvement (β = −0.25; 95% CI, −0.59 to 0.10; *P* = .17) or worsening (β = 0.18; 95% CI, −0.22 to 0.59; *P* = .38) with HbA_1c_ levels among all participants.

**Conclusions and Relevance:**

In this secondary analysis of data from a randomized clinical trial, an MCID improvement or worsening of more than 0.25 on the DDS-17 was quantitatively significant and patients in the EPICC group were more likely to experience improvement than those in the EUC group.

**Trial Registration:**

ClinicalTrials.gov Identifier: NCT01876485

## Key Points

**Question:**

What are the minimal clinically important differences (MCIDs) in the Diabetes Distress Scale–17 (DDS-17) and its 4 subscales?

**Findings:**

This secondary analysis using data from 248 participants in a randomized clinical trial comparing the Empowering Patients in Chronic Care (EPICC) intervention (123 participants) with enhanced usual care (EUC; 125 participants) found that the overall MCID value for DDS-17 was 0.25, and MCIDs were 0.38 for emotional and interpersonal distress subscales and 0.39 for physician and regimen distress subscales. Participants in the EPICC group were more likely to have significant improvements and less likely to have significant declines in DDS-17 compared with participants in EUC.

**Meaning:**

These findings suggest that MCID changes of 0.25 or greater were associated with clinically important improvements in diabetes distress.

## Teaser

This secondary analysis uses randomized clinical trial data to establish a distribution-based metric for minimal clinically important differences in Diabetes Distress Scale–17 scores and assesses whether changes on these levels are associated with changes in glycated hemoglobin management.

## Introduction

Clinical trials demonstrate lower morbidity and mortality in patients with type 2 diabetes by reducing hemoglobin A_1c_ (HbA_1c_) levels.^1^ Because diabetes is a chronic condition, sustained reduction of HbA_1c_ requires patient activation, commitment to treatment planning, and self-management.^2^ The lifestyle changes required to manage diabetes may carry an emotional burden that contributes to diabetes-associated distress.^3^ Diabetes distress refers to the worries, fears, and threats arising from struggles with chronic diabetes care (ie, management, complications, and loss of function)^4^ and is associated with changes in HbA_1c_ levels.^5,6^ Patients with high distress have significantly higher HbA_1c_ levels and are less likely to maintain blood glucose levels within the reference range.^7^

The Diabetes Distress Scale–17 (DDS-17) is an established, validated measure with 17 items to assess the level of distress in patients with diabetes.^8,9,10^ Higher DDS-17 scores are associated with poor lifestyle choices, self-management, self-efficacy, self-care, and adherence to recommended treatment regimens,^11,12,13^ while lower scores are associated with reductions in HbA_1c_.^14^ Prior DDS-17 validation studies have suggested severity thresholds as little or no distress, less than 2.0; moderate distress, 2.0 to 2.9; and high distress, greater than 3.0.^15^ DDS-17 is often used as a dichotomous variable, with scores of 2.0 or greater signifying the presence of moderate diabetes distress.^11,13,15^ However, cutpoints are limited by their inability to capture significant changes in DDS-17 scores that do not cross a cutpoint. For example, an individual whose DDS-17 score decreases from 2.8 to 2.1 may experience meaningful improvements in diabetes distress, but the moderate distress cutoff is unchanged. This limitation can be overcome through calculation of minimal clinically important differences (MCIDs). MCIDs are useful in interpreting the clinical relevance of observed changes at both individual and group levels.^9,10^ Given that DDS-17 is scored on a continuous scale, distribution-based MCIDs are a useful alternative to dichotomous cutoff scores. Distribution-based MCIDs are defined as a numerical score that represents the smallest value of change anywhere along the entire range of a continuous measure that would be considered meaningful.^8,10^

We previously developed Empowering Patients in Chronic Care (EPICC) and described its value in a series of studies.^16,17,18,19,20,21,22,23,24^ EPICC is a goal-setting intervention that uses coaching and motivational interviewing to activate patients to explore what matters most to them about their health,^16,17^ set outcome goals based on their priorities,^18,19^ develop skills to communicate goals with clinicians,^20^ and create action plans to achieve their goals.^21,22^ EPICC has been successfully adopted into the routine primary care workflow using implementation strategies.^23^ A 2022 multisite clinical trial^24^ demonstrated the effectiveness of EPICC compared with enhanced usual care (EUC) at reducing HbA_1c_ and diabetes distress 4 months after the intervention in routine primary care practices. Diabetes distress was assessed in the EPICC trial using the DDS-17.

In this study, we establish the distribution-based MCIDs for DDS-17 and each of the 4 subscales of the DDS-17 using a quantitative calculation translated into 3 categories of change in DDS-17 scores: improvement, no change, and worsening. We then compare the percentage identified in each MCID category relative to the percentage of participants defined as changing based on crossing over the established DDS-17 cutpoint of 2.0. We also examined associations of MCID categories with participation in the EPICC treatment group and change in HbA_1c_ levels.

## Methods

This secondary analysis of a randomized clinical trial was approved by the Department of Veterans Affairs (VA) central institutional review board, and each clinic-based research and development committee approved the protocol. All participants provided verbal informed consent by telephone. This study reports on secondary outcomes from a multisite, randomized clinical trial of the EPICC intervention conducted from July 1, 2015, through June 30, 2017, among participants with treated but uncontrolled diabetes.^24,25^ The study conformed to the Consolidated Standards of Reporting Trials (CONSORT) reporting guideline. The trial protocol and statistical analysis plan are provided in Supplement 1.

### Study Design and Participants

We previously described the intervention protocol and primary results of the EPICC study.^24,25^ In that study, we used a hybrid (implementation-effectiveness) clinical trial design to randomize 280 participants to EPICC or EUC in VA primary care clinics across Illinois, Indiana, and Texas. The inclusion criterion was a diagnosis of uncontrolled diabetes with a mean HbA_1c_ level greater than 8.0% (to convert to proportion of total hemoglobin, multiply by 0.01) in the prior 6 months. EUC participants received routine care that included educational materials, nutrition counseling, medication management or weight loss support, a list of self-management resources routinely offered at their site (eg, traditional diabetes education). EPICC participants attended 6 bimonthly group sessions for 50 minutes each, followed by 10-minute 1-on-1 sessions based on collaborative goal setting and motivational interviewing theory for 3 months. The trial’s primary outcomes evaluated the clinical effectiveness of EPICC compared with EUC after the intervention.^24,25^

### Measures and Scales Used

Diabetes distress was measured in this study using the DDS-17. The DDS-17 consists of 17 items that measure patients’ perceptions in 4 general domains of distress: interpersonal, physician, regimen, and emotional. Interpersonal distress (3 items) reflects the psychological emotions and feelings of patients with diabetes during their interaction with people around them. Physician distress (4 items) portrays the distress that patients experience during interaction with their physician. Regimen distress (5 items) describes the distress felt by patients because of the need to adhere to a diabetes management plan. Emotional burden (5 items) describes the distress related to emotions associated with having diabetes.^26^ Each individual item is measured on a Likert scale of 1 (no distress) to 6 (serious distress), and a mean composite score is also determined, with higher scores indicating greater distress.^27,28^ The DDS-17 has been validated across a number of settings for assessing distress levels.^11,12,13,14,29^ Both the total DDS-17 and its subscales demonstrate good internal consistency, reliability, and construct validity, given associations with depression measures, metabolic variables, and disease management, as well as lack of associations with sex, ethnicity, and education.^11,29^ This study includes the 248 individuals from the EPICC trial who have DDS-17 and HbA_1c_ data at both baseline and postintervention (4 months after baseline) assessments.

### Statistical Analysis

We first used independent samples *t* tests and χ^2^ tests to evaluate whether participants who completed DDS-17 at both baseline (248 participants) and the postintervention assessment differed from those who only completed the baseline assessment (32 participants). We then calculated descriptive statistics (means, SDs, frequencies) for demographic characteristics overall and separately for each treatment group. Race and ethnicity were collected through self-report and categorized as Hispanic, non-Hispanic Black, non-Hispanic White, and other (including American Indian and other race or ethnicity not specified). Race and ethnicity data were included in analyses to maximize data richness and minimize opportunities for researchers’ assumptions about participants’ identities.

#### Calculation of MCID and MCID Change Categories

MCIDs can be calculated using distribution-based approaches. Distribution-based approaches are based on statistical criteria from patient-reported outcome scores.^30^ These approaches include fractions of the SD of patient-reported outcome scores, the effect size,^31^ and the standard error of measurement (SEM)^8,32^ as estimates for the MCID. A score change greater than or equal to the value of the SEM represents meaningful variation in the measured construct that is likely not due to measurement error.^10^ This method produces MCIDs that are expressed in the same units of measurement as the patient-reported outcome score.^32^ We used the SEM distribution-based method, which uses the SD and Cronbach α of baseline scores, SD × sqrt(1 − α)^8^ to calculate the MCID for the DDS-17 and its 4 subscales.

Using the resulting DDS-17 MCID value, we determined whether change on the DDS-17 and each of 4 subscales from baseline to after the intervention indicated improvement (a decrease ≥ the MCID value), no change (stayed within ± the MCID value), or worsening (an increase ≥ the MCID value). Given prior validation of the DDS-17 cutpoint of 2 indicating moderate distress,^15^ we evaluate 3 categories of change between baseline and after the intervention across this cutpoint: (1) participants who started with scores greater than 2 at baseline and crossed to less than 2, (2) participants who started with scores less than 2 at baseline and crossed to greater than 2, and (3) participants who did not cross the cutpoint from baseline to after the intervention.

#### Association Between Treatment Group and MCID Change Category

Given participants were nested within cohorts that were also nested within sites, we calculated intraclass correlation coefficients (ICC) for the total DDS-17 as well as the 4 subscales to determine whether multilevel models accounting for dependency in the data were warranted. The degree of variance in the DDS-17 attributable to differences between both cohort and site (ie, ICCs >0.05), indicated that multilevel models accounting for the dependency of participants (level 1) within cohorts (level 2) within sites (level 3) were warranted. For the DDS-17 and each of the 4 subscales, 2 sets of multilevel logistic regression models were used to evaluate differences between EPICC and EUC participants in DDS-17 MCID categories. The first set consisted of an examination of treatment group (in which EPICC = 1 and EUC = 0) as a factor of whether a participant showed MCID improvement (with yes = 1 and no = 0, which collapsed no change and worsening) and the second set consisted of an examination of treatment group as a factor of whether a participant showed MCID worsening (with 1 = yes and 0 = no, which collapsed no change and improvement). For each set, 5 models were conducted: 1 for DDS-17 and the 4 subscales. Prior diabetes education was included as a covariate in all models examining differences between treatment groups, given differences between treatment groups in this variable.

#### Association Between DDS-17 MCID Change Category and HbA_1c_

Change in HbA_1c_ was calculated by subtracting baseline scores from postintervention scores, such that negative values indicated reduction (clinical improvement) in HbA_1c_ values. We first calculated descriptive statistics to evaluate mean change in HbA_1c_ by MCID improvement, worsening, and no change. We subsequently used a pair of multilevel linear regression models for each the DDS-17 and the 4 subscales to examine the effect of MCID category on change in HbA_1c_ values from baseline to after the intervention. The first set of models examined MCID improvement (with yes = 1 and no = 0) as a factor and the second set examined MCID worsening (with yes = 1 and no = 0) as a factor. Treatment group was included as a covariate in all models. Analyses were conducted using SAS version 9.4 (SAS Institute). Sample SAS code for our analyses is provided in the eMethods in Supplement 2. *P* values were 2-tailed, and statistical significance was set at α = .05. Data collection was completed in November 2018, and data analysis was completed in June 2023.

## Results

### Participant Characteristics

A total of 248 individuals with complete DDS-17 data were included (mean [SD] age, 67.4 [8.3] years; 235 [94.76%] men), with 123 participants in the EPICC group and 125 participants in the EUC group (Table 1). There were 28 Hispanic participants (11.30%), 94 non-Hispanic Black participants (37.90%), and 121 non-Hispanic White participants (48.79%). The 32 participants without postintervention DDS-17 data did not significantly differ on any demographics or baseline characteristics from the 248 participants with postintervention DDS-17 data. Therefore, we proceeded to calculate MCID values and subsequent analyses among the 248 participants with DDS-17 scores at both assessments (Figure). Most participants had an annual income of less than $40 000 (143 participants [62.17%]) and had at least some college education (185 participants [74.60%]).

**Table 1.** Treatment Group Demographics and Baseline Clinical Characteristics Overall and by Treatment Group

Characteristic	Participants, No. (%)		
	Total (n = 248)^a^	EPICC (n = 123)	EUC (n = 125)
Age, mean (SD), y	67.35 (8.30)	67.69 (8.66)	67.02 (7.96)
Sex			
Male	235 (94.76)	117 (95.12)	118 (94.40)
Female	13 (5.24)	6 (4.88)	7 (5.60)
Race/ethnicity			
Hispanic	28 (11.29)	19 (15.45)	9 (7.20)
Non-Hispanic Black	94 (37.90)	40 (32.52)	54 (43.20)
Non-Hispanic White	121 (48.79)	63 (51.22)	58 (46.40)
Other^a^	5 (2.02)	1 (0.81)	4 (3.20)
Education			
≤High school	63 (25.40)	35 (28.46)	28 (22.40)
≥Some college	185 (74.60)	88 (71.54)	97 (77.60)
Annual income (n = 230)			
<$20 000	71 (30.87)	37 (32.17)	34 (29.57)
$20 000-$39 999	72 (31.30)	36 (31.30)	36 (31.30)
>$40 000	87 (37.83)	42 (36.52)	45 (39.13)
Employment (n = 241)			
Any employment	37 (15.35)	16 (13.56)	21 (17.07)
Unemployed	15 (6.22)	6 (5.08)	9 (7.32)
Retired or disabled	189 (78.42)	96 (81.36)	93 (75.61)
Prior diabetes education	143 (57.66)	62 (50.41)	81 (64.80)
HbA_1c_ level, mean (SD), %	9.07 (1.43)	9.04 (8.77)	9.10 (8.87)

Abbreviations: EPICC, Empowering Patients in Chronic Care; EUC, enhanced usual care; HbA_1c_, hemoglobin A_1c_.

SI conversion factor: To convert HbA_1c_ to proportion of total hemoglobin, multiply by 0.01.

^a^ Includes multiracial (eg, 3 participants identified as American Indian, White, and other endorsed), other – not specified (2 participants). Data on race and ethnicity are self-reported.

**Figure.**
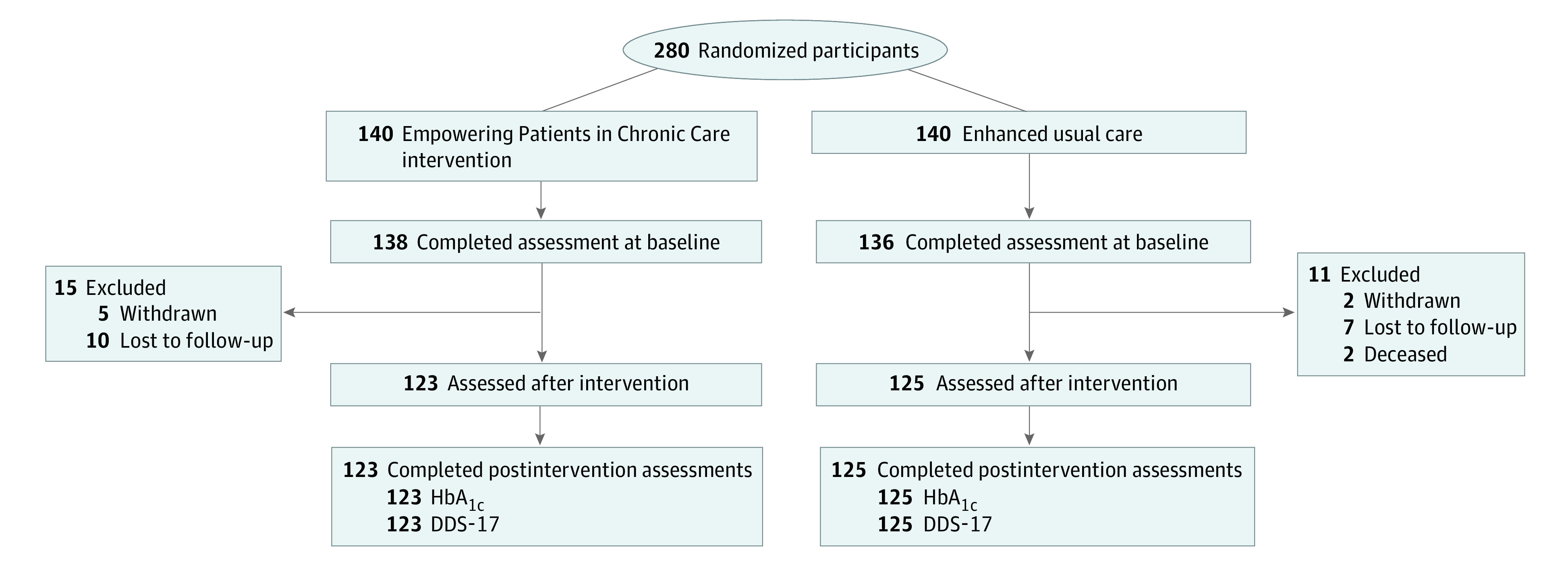
Participant Inclusion Flowchart DDS-17 indicates Diabetes Distress Scale–17; HbA_1c_, glycated hemoglobin A_1c_.

### Distribution-Based MCID Values and Change Categories

The MCID for DDS-17 was 0.25, with subscale MCID values of 0.38 for emotional distress and interpersonal distress and 0.39 for physician distress and regimen distress (Table 2). MCID captured a different degree of change compared with the DDS-17 cutoff level of 2.0. From baseline to postintervention, 103 participants (41.53%) experienced improvement (≥0.25 decrease in DDS-17), 59 participants (23.79%) experienced worsening (≥0.25 increase in DDS-17), and 86 participants (34.68%) had no change, ie, their change was less than 0.25 on the DDS-17. In comparison, only 49 participants (19.76%) of all participants with DDS-17 levels greater than 2 at baseline reported scores that decreased less than 2 after the intervention. Only 25 participants (10.08%) with DDS-17 levels less than 2 at baseline reported scores that increased to greater than 2 after the intervention. Most participants remained either above (102 participants [41.13%]) or below (72 participants [29.03%]) the DDS-17 cutoff of 2 during both study time points. For DDS-17 subscales, MCID improvements were reported by 107 participants (43.15%) for emotional distress, 62 participants (25.00%) for physician distress, 119 participants (47.98%) for regimen distress, and 65 participants (26.21%) for interpersonal distress among all participants.

**Table 2.** Distribution-Based MCID Calculations for the Diabetes Distress Scale

Measure	No. of scale items	Baseline mean (SD) [95% CI]	Baseline α	Distribution-based MCID
Total diabetes distress	17	2.40 (1.03) [2.27-2.53]	0.94	0.25
Emotional distress	5	2.65 (1.25) [2.49-2.81]	0.91	0.38
Physician distress	4	1.85 (1.17) [1.71-2.00]	0.89	0.39
Regimen distress	5	2.81 (1.23) [2.66-2.96]	0.90	0.39
Interpersonal distress	3	2.02 (1.25) [1.86-2.18]	0.91	0.38

Abbreviation: MCID, minimal clinically important differences.

### Treatment Group and MCID Change Categories

A greater proportion of participants in the EPICC cohort reported an MCID improvement compared with participants in EUC (63 participants [51.22%] vs 40 participants [32.00%]) **(**Table 3). EPICC participants were significantly more likely to be in the improved category for DDS-17 overall (odds ratio [OR], 2.24 [95% CI, 1.33 to 3.78]) and for emotional distress (OR, 2.24 [95% CI, 1.33 to 3.77]) and regimen distress (OR, 1.86 [95% CI, 1.11 to 3.12]) subscales compared with EUC participants. Treatment group was unrelated to DDS-17 MCID improvement for physician distress and interpersonal distress. Participants who received EPICC were significantly less likely to be in the MCID worsening category for DDS-17 overall (OR, 0.43 [95% CI, 0.23 to 0.80]), regimen distress (OR, 0.41 [95% CI, 0.22 to 0.77]), and interpersonal distress (OR, 0.46 [95% CI, 0.24 to 0.89) scores compared with EUC participants. Treatment group was unrelated to DDS-17 MCID worsening for emotional distress and physician distress (Table 3).

**Table 3.** Frequency of Each DDS-17 MCID Change Category and the Effect of Treatment Group on MCID Change Category

Measure	MCID category, No. (%)^a^			Association of treatment group with MCID category^b^				
	Significant improvement	No change	Significant worsening	Significant improvement		Significant worsening		
				Odds ratio (95% CI)^c^	*P* value	OR (95% CI)^c^	*P* value
**Total DDS-17**								
Total	103 (41.53)	86 (34.68)	59 (23.79)	2.24 (1.33-3.78)	.003	0.43 (0.23-0.80)	.008
EPICC	63 (51.22)	40 (32.52)	20 (16.26)				
EUC	40 (32.00)	46 (36.80)	39 (31.20)				
**Emotional distress**								
Total	107 (43.15)	84 (33.87)	57 (22.98)	2.24 (1.33-3.77)	.003	0.65 (0.35-1.20)	.17
EPICC	65 (52.85)	35 (28.46)	23 (18.70)				
EUC	42 (33.60)	49 (39.20)	34 (27.20)				
**Physician distress**								
Total	62 (25.00)	139 (56.05)	47 (18.95)	1.15 (0.63-2.10)	.64	0.66 (0.34-1.27)	.21
EPICC	34 (27.64)	70 (56.91)	19 (15.45)				
EUC	28 (22.40)	69 (55.20)	28 (22.40)				
**Regimen distress**								
Total	119 (47.98)	70 (28.23)	59 (23.79)	1.86 (1.11-3.12)	.02	0.41 (0.22-0.77)	.006
EPICC	69 (56.10)	34 (27.64)	20 (16.26)				
EUC	50 (40.00)	36 (28.80)	39 (31.20)				
**Interpersonal distress**								
Total	65 (26.21)	133 (53.63)	50 (20.16)	1.34 (0.74-2.43)	.33	0.46 (0.24-0.89)	.02
EPICC	36 (29.27)	70 (56.91)	17 (13.82)				
EUC	29 (23.20)	63 (50.40)	33 (26.40)				

Abbreviations: DDS-17, Diabetes Distress Scale–17; EPICC, Empowering Patients in Chronic Care; EUC, enhanced usual care; MCID, minimal clinically important differences.

^a^ Change was from baseline to after the intervention. Significant worsening indicates an increase in DDS-17 Score by the MCID for total DDS-17 and each subscale. Significant improvement indicates a decrease in DDS-17 score by the MCID for total DDS-17 and each subscale.

^b^ All models account for dependency of participants within cohorts and sites and control for prior diabetes education.

^c^ Values greater than 1 indicate the event is more likely; less than 1, less likely.

### Change in HbA_1c_ Levels by DDS-17 MCID Categories

Mean reduction in HbA_1c_ from baseline to after the intervention was higher among the total DDS-17 MCID improvement category (−0.44% [95% CI, −0.74% to −0.14%]), compared with the no change (−0.17% [95% CI, −0.39% to 0.05%]) and worsening (−0.06% [95% CI, −0.39% to 0.27%]) categories (Table 4). However, neither DDS-17 MCID improvement nor worsening categories were associated with significant change in HbA_1c_ scores (improvement: β = −0.25 [95% CI, −0.59 to 0.10]; *P* = .17; worsening: β = 0.18 [95% CI, −0.22 to 0.59]; *P* = .38). There were no significant associations for DDS-17 MCID improvement or worsening categories on HbA_1c_ change among the overall sample.

**Table 4.** Mean Change in HbA_1c_ by DDS-17 MCID Change Category and Associations of MCID Improvement and Worsening With Change in HbA_1c_

DDS-17	MCID change category, HbA_1c_ change %, mean (95% CI)			Multilevel linear regression models estimating change in HbA_1c_			
	Improvement (n = 103)	No change (n = 86)	Worsening (n = 59)	MCID improvement		MCID worsening	
				β (95% CI)^a^	*P* value	β (95% CI)^a^	*P* value
Total	−0.44 (−0.74 to −0.14)	−0.17 (−0.39 to 0.05)	−0.06 (−0.39 to 0.27)	−0.25 (−0.59 to 0.10)	.17	0.18 (−0.22 to 0.59)	.38
Emotional distress subscale	−0.27 (−0.50 to −0.04)	−0.20 (−0.55 to 0.15)	−0.31 (−0.60 to −0.022)	0.05 (−0.30 to 0.40)	.77	−0.10 (−0.50 to 0.30)	.62
Physician distress subscale	−0.38 (−0.64 to −0.13)	−0.25 (−0.54 to 0.04)	−0.12 (−0.46 to 0.22)	−0.17 (−0.57 to 0.24)	.43	0.13 (−0.31 to 0.56)	.56
Regimen distress subscale	−0.45 (−0.75 to −0.15)	−0.20 (−0.40 to 0.003)	0.08 (−0.24 to 0.40)	−0.35 (−0.68 to 0)	.05	0.37 (−0.04 to 0.77)	.08
Interpersonal distress subscale	−0.36 (−0.61 to −0.11)	−0.21 (−0.51 to 0.09)	−0.26 (−0.57 to 0.05)	−0.12 (−0.51 to 0.27)	.54	−0.08 (−0.51 to 0.34)	.70

Abbreviations: DDS-17, Diabetes Distress Scale–17; HbA_1c_, hemoglobin A_1c_; MCID, minimal clinically important differences.

^a^ Values greater than 0 indicate the event is more likely; less than 0, less likely.

## Discussion

This secondary analysis of a randomized clinical trial established an MCID value of 0.25 for the DDS-17, 0.38 MCID for emotional distress and regimen distress subscales, and 0.39 MCID for physician distress and regimen distress subscales. Distribution-based MCIDs are a numerical score that represents the smallest value of change that would be considered meaningful anywhere along the entire range of a continuous measure. These values provide ranges for defining significant improvement (≥0.25 decline in DDS-17), no change (DDS-17 change of ≤0.25), and significant worsening (≥0.25 increase in DDS-17) in diabetes distress levels. Participants in the EPICC intervention were significantly more likely to be in the improving category and less likely to be in the worsening category. MCID improvement in the DDS-17 was associated with mean HbA_1c_ reduction of 0.44%. However, no statistically significant associations were found between HbA_1c_ change and MCID improvement or worsening in the DDS-17. No subscale had statistically significant associations of MCID change with HbA_1c_ change. Previous research has provided evidence for an association among regimen distress, behavioral self-management, and glycemic control, positing that improvements in management and HbA_1c_ levels co-occur with improvements with regimen distress.^33^ This prior work, coupled with our findings, provide support for addressing regimen distress in clinical care as part of diabetes management.

This study was the first, to our knowledge, to calculate the MCID for the DDS-17 and each of the 4 subscales of the DDS-17. A combination of both anchor- and distribution-based methods is typically perceived as the preferred method for calculating MCIDs.^34^ The anchor-based option was not applicable for our calculation of MCID, since we did not ask participants to quantify the extent to which they felt their diabetes distress changed from baseline to after the intervention. The distribution-based MCID values calculated in this study (0.25 to 0.39) closely align with previous research defining the MCID for the 28-item T1-Diabetes Distress Scale and its subscales (0.19 to 0.50).^8^ MCID values of the Type 2 Diabetes Distress Assessment have also recently been defined (0.25) and was similar to the DDS-17 MCID score calculated in this study, indicating consistency across similar diabetes distress scales.^35^ In this study, we establish an MCID change of at least 0.25 as a quantitative metric for determining clinically important change in DDS-17 scores. This provides pragmatic guidance for intervention studies that complements the established DDS-17 cutoff score of 2.0 previously described in literature.^11,13,15^

For the DDS-17 and its 4 subscales, we used 3 classifications to characterize change in scores from baseline to after the intervention: MCID improvement, no change, or MCID worsening. This approach adds to the binary (yes vs no) improvement concept by introducing the clinically important state of not worsening. Given the heterogeneous patterns of associations between DDS-17 MCID categories and treatment group, categorizing change in DDS-17 scores as improvement (yes vs no) or worsening (yes vs no) relative to their MCID value may indicate dual ways to frame response to a treatment: improvement or not worsening. Quantitative trends in HbA_1c_ change were observed among the MCID worsening, no change, and improvement categories. However, this association was not statistically significant. These findings suggest that significant change in HbA_1c_ may require greater than MCID levels of improvement in diabetes distress.

### Limitations

The study has limitations. Results may be limited to a population of largely male veterans seen in primary care clinics within the VA. However, MCID results used an established methodology found in prior studies calculating MCID for other diabetes distress scales with corresponding MCID values. MCID values were calculated using a distribution method only, which maybe limited without a corresponding anchor value that provides a subjective measure of change from a baseline.^36,37^ Given that we did not ask participants how their diabetes distress changed from baseline to after the intervention (ie, worsened, no change, improved), an anchor-based method was not possible for us to use. In using the SEM distribution-based method, we did allow the MCIDs calculated to be better applied to diverse populations as the SEM is a property of the scale, not a property of a particular sample’s DDS-17 distribution.^8^ Analyses were limited to data collected during a 4-month period using 2 assessments as part of a clinical trial. However, participants of the current study were recruited from a large, diverse, community sample of adults with diabetes across 3 states. Data from longitudinal cohort studies outside of an intervention trial may be needed to replicate and extend our findings.

## Conclusions

This secondary analysis of a randomized clinical trial identified improvement or worsening of at least 0.25 on the DDS-17 scale as the MCID. This MCID value is an appropriate method for assessing significant change in the DDS-17 from baseline to after a treatment intervention, given the evidence for an association between MCID improvements in DDS-17 scores among EPICC participants. The MCID values identified in this study can be used to inform future research examining diabetes distress using the DDS-17. Further, MCID values for DDS-17 can potentially be used by clinicians to assess response to treatments in their patients.

## Notes

Supplement 1.
Trial Protocol and Statistical Analysis Plan

Supplement 2.
eMethods

Supplement 3.
Data Sharing Statement
