# Supplementary material for: Ascertainment of Minimal Clinically Important Differences in the Diabetes Distress Scale–17: A Secondary Analysis of a Randomized Clinical Trial
Source: JAMA Netw Open. 2023 Nov 15;6(11):e2342950. doi: 10.1001/jamanetworkopen.2023.42950 (PMC10652154; doi:10.1001/jamanetworkopen.2023.42950)
Supplement: Supplement 2. — eMethods [file jamanetwopen-e2342950-s002.pdf]

Supplemental Online Content

Banks J, Amspoker AB, Vaughan EM, Woodard L, Naik AD. Ascertainment of minimal clinically important differences in the Diabetes Distress Scale–17: a secondary analysis of a randomized clinical trial. JAMA Netw Open. 2023;6(11):e2342950. doi:10.1001/jamanetworkopen.2023.42950

eMethods.

This supplemental material has been provided by the authors to give readers additional information about their work.

© 2023 Banks J et al. *JAMA Network Open.*

eMethods

SAS Version 9.4 code to accompany primary analyses

Determination of total DDS-17 MCID category membership based on change in DDS-17 scores from baseline to post-intervention

```
data MCIDcategory
set onlythere;
if chgdds_total <= -0.25 then MCID_category = 'improved';
if chgdds_total > -0.25 and chgdds_total < 0.25 then MCID_category = 'no change';
if chgdds_total >= 0.25 then MCID_category = 'worsened';
run;
```

Generalized linear mixed model examining treatment arm predicting improvement in total DDS-17 MCID (improve = 1, worsen/no change = 0)

```
proc glimmix data = MCIDcategory;
class cohort site prior_dm_education (ref = first) treatmentdummy (ref = first);
model MCIDimprove (descending) = prior_dm_education treatmentdummy / dist = binary
link = logit solution oddsratio;
random intercept / sub = site type = un;
random intercept / sub = cohort(site) type = un;
run;
```

Generalized linear mixed model examining treatment arm predicting worsening in total DDS-17 MCID (worsen = 1, improve/no change = 0)

```
proc glimmix data = MCIDcategory;
class cohort site prior_dm_education (ref = first) treatmentdummy (ref = first);
model MCIDworsen (descending) = prior_dm_education treatmentdummy / dist = binary link
= logit solution oddsratio;
random intercept / sub = site;
random intercept / sub = cohort(site);
run;
```

Linear mixed model examining total DDS-17 MCID improvement predicting change in HbA1c from baseline to post-intervention

```
proc mixed data = MCIDcategory;
class cohort site prior_dm_education treatmentdummy;
model ChgHba1c = prior_dm_education treatmentdummy MCIDimprove /solution ddfm = bw;
random intercept / sub = site;
random intercept / sub = cohort(site);
run;
```

Linear mixed model examining total DDS-17 MCID improvement predicting change in HbA1c from baseline to post-intervention

```
proc mixed data = MCIDcategory noclprint covtest;
class cohort site prior_dm_education treatmentdummy;
model ChgHba1c = prior_dm_education treatmentdummy MCIDworsen /solution ddfm = bw;
random intercept / sub = site;
random intercept / sub = cohort(site);
run;
```

© 2023 Banks J et al. *JAMA Network Open.*
