# Supplementary material for: Ascertainment of Minimal Clinically Important Differences in the Diabetes Distress Scale–17: A Secondary Analysis of a Randomized Clinical Trial
Source: JAMA Netw Open. 2023 Nov 15;6(11):e2342950. doi: 10.1001/jamanetworkopen.2023.42950 (PMC10652154; doi:10.1001/jamanetworkopen.2023.42950)
Supplement: Supplement 3. — Data Sharing Statement [file jamanetwopen-e2342950-s003.pdf]

Data Sharing Statement

Banks. Ascertainment of Minimal Clinically Important Differences in the Diabetes Distress Scale–17. *JAMA Netw Open*. Published November 15, 2023. doi:10.1001/jamanetworkopen.2023.42950

Data
Data available: No
